# Extracellular Release and Signaling by Heat Shock Protein 27: Role in Modifying Vascular Inflammation

**DOI:** 10.3389/fimmu.2016.00285

**Published:** 2016-07-26

**Authors:** Zarah Batulan, Vivek Krishna Pulakazhi Venu, Yumei Li, Geremy Koumbadinga, Daiana Gisela Alvarez-Olmedo, Chunhua Shi, Edward R. O’Brien

**Affiliations:** ^1^Vascular Biology Laboratory, Health Research Innovation Centre, Libin Cardiovascular Institute of Alberta, University of Calgary Cumming School of Medicine, Calgary, AB, Canada; ^2^Oncology Laboratory, Institute for Experimental Medicine and Biology of Cuyo (IMBECU), CCT CONICET, Mendoza, Argentina

**Keywords:** heat shock protein 27, inflammation, atherosclerosis, coronary artery disease, HSPB1, non-classical secretion, extracellular HSP27

## Abstract

Heat shock protein 27 (HSP27) is traditionally viewed as an intracellular chaperone protein with anti-apoptotic properties. However, recent data indicate that a number of heat shock proteins, including HSP27, are also found in the extracellular space where they may signal *via* membrane receptors to alter gene transcription and cellular function. Therefore, there is increasing interest in better understanding how HSP27 is released from cells, its levels and composition in the extracellular space, and the cognate cell membrane receptors involved in effecting cell signaling. In this paper, the knowledge to date, as well as some emerging paradigms about the extracellular function of HSP27 is presented. Of particular interest is the role of HSP27 in attenuating atherogenesis by modifying lipid uptake and inflammation in the plaque. Moreover, the abundance of HSP27 in serum is an emerging new biomarker for ischemic events. Finally, HSP27 replacement therapy may represent a novel therapeutic opportunity for chronic inflammatory disorders, such as atherosclerosis.

## Introduction

Since its first discovery in 1962 by Ritossa, the heat shock response has been established as the classical molecular mechanism underlying the cellular response to heat stress, involving the upregulation of a spectrum of protein families designated as “heat shock proteins” (HSPs) (1, 2). These proteins range in molecular weight from 10 to 110 kDa and primarily function as “chaperones” that facilitate proper protein folding (3, 4). Exposed hydrophobic residues in nascent or misfolded proteins act as the signal to recruit and activate HSPs, making this class of proteins powerful sensors of environmental (e.g., heat, oxidative) and physiological (e.g., infection, inflammation) stress (5, 6). Although initially characterized as *intracellular* chaperones, subsequent studies pointed to the presence of HSPs in the *extracellular* space (7, 8). Extracellular HSPs (eHSPs) have consequently been ascribed novel functions (such as “outside–in” signaling leading to cellular proliferation and immune response modulation), expanding their canonical role of maintaining homeostasis in the cell to that of the whole organism.

## Intracellular Role of HSP27

Unlike the larger molecular weight HSPs, HSP27 was first identified as an estrogen-responsive protein (9–11), which was later confirmed to be heat-inducible and sharing sequence homology with other HSPs (12–16). It is a member of the small heat shock protein beta (HSPB) family, an HSP subgroup characterized by a lower molecular weight range (12–43 kDa) and a shared α-crystallin domain flanked by variable N- and C-terminal sequences (17, 18). The encoding gene, *HSPB1*, is located on chromosome 7 (7q11.23) (19), and its associated mutations have been linked to Charcot–Marie–Tooth disease type 2 (20, 21) and possibly Williams syndrome (22, 23). The intracellular chaperoning function of HSP27 is largely regulated by the phosphorylation/dephosphorylation of three key N-terminal serine residues at positions 15, 78, and 82 (24–26), which influences the assembly of HSP27 monomers into large oligomeric complexes (200–800 kDa). Such complexes create an ATP-independent chaperone network that effectively traps misfolded protein substrates, preventing their aggregation through a series of controlled binding and release reactions that, in conjunction with other larger HSPs (HSP70, HSP90), facilitate their correct folding (27–29). Thus, in concert with other chaperones, HSP27 maintains cellular homeostasis by holding misfolded proteins in a soluble, refolding-competent form. Intracellular HSP27 has also been implicated in (i) cytoskeletal architecture and dynamics (30–32), (ii) mRNA stabilization (33), (iii) antioxidant responses (34–36), and (iv) anti-apoptosis (37–40). Not surprisingly, its multiple, cytoprotective effects are associated with the amelioration of a range of pathological conditions, including degenerative, ischemic, neurological, and cardiovascular disease (41). For more comprehensive reviews on the biology of intracellular HSP27, several references are available (17, 42–44).

## Extracellular Role of HSP27

Heat shock proteins were first observed in the extracellular space nearly 30 years ago as proteins that were transferred from glia to neurons in the squid giant axon (45) and later confirmed to be released after heat shock in mammalian cells (46). Like other HSPs, HSP27 has been found extracellularly – initial evidence indicated its secretion from tumor cells (47–51). The presence of HSP27 has been detected in human serum, with elevations observed after extreme exercise (52, 53). Increases in serum HSP27 have also been measured in patients with chronic pancreatitis (54), gastric adenocarcinoma (55), insulin resistance (56), and during acute attacks in multiple sclerosis (57), while decreases have been associated with type 1 diabetes (58). HSP27 has also been found in the cerebrospinal fluid during brain and spinal cord ischemia (59).

In the context of cardiovascular disease, two independent groups utilizing a proteomic analysis of human atherogenic arterial tissue have shown that HSP27 is differentially secreted into the extracellular milieu (60, 61). Moreover, serum levels of HSP27 have been detected in patients with atherosclerosis (60, 62, 63), acute coronary syndromes (61), and reperfusion after ischemic clamping during heart bypass surgery (64). The presence of serum HSP27 was confirmed in an atherosclerosis mouse model (ApoE^−/−^), overexpressing HSP27 (HSP27^o/e^) using commercially available ELISA kits (65, 66). However, these experiments revealed the presence of HSP27-reactive autoantibodies (AAB) in the serum that effectively shield the detection of HSP27, thereby resulting in erroneously low levels of HSP27. Consequently, our laboratory has developed a mass spectrometric-based method that can more sensitively detect serum HSP27 at levels higher than those quantified using commercial ELISA kits (67). Even though the detection of serum HSP27 levels can only improve, results to date firmly establish the extracellular presence of HSP27. However, key questions remain (1) how is HSP27 released from cells; (2) what is its function outside the cell; and (3) how does it signal from outside to inside the cell?

## Possible Mechanisms of HSP27 Export

**Figure 1:**
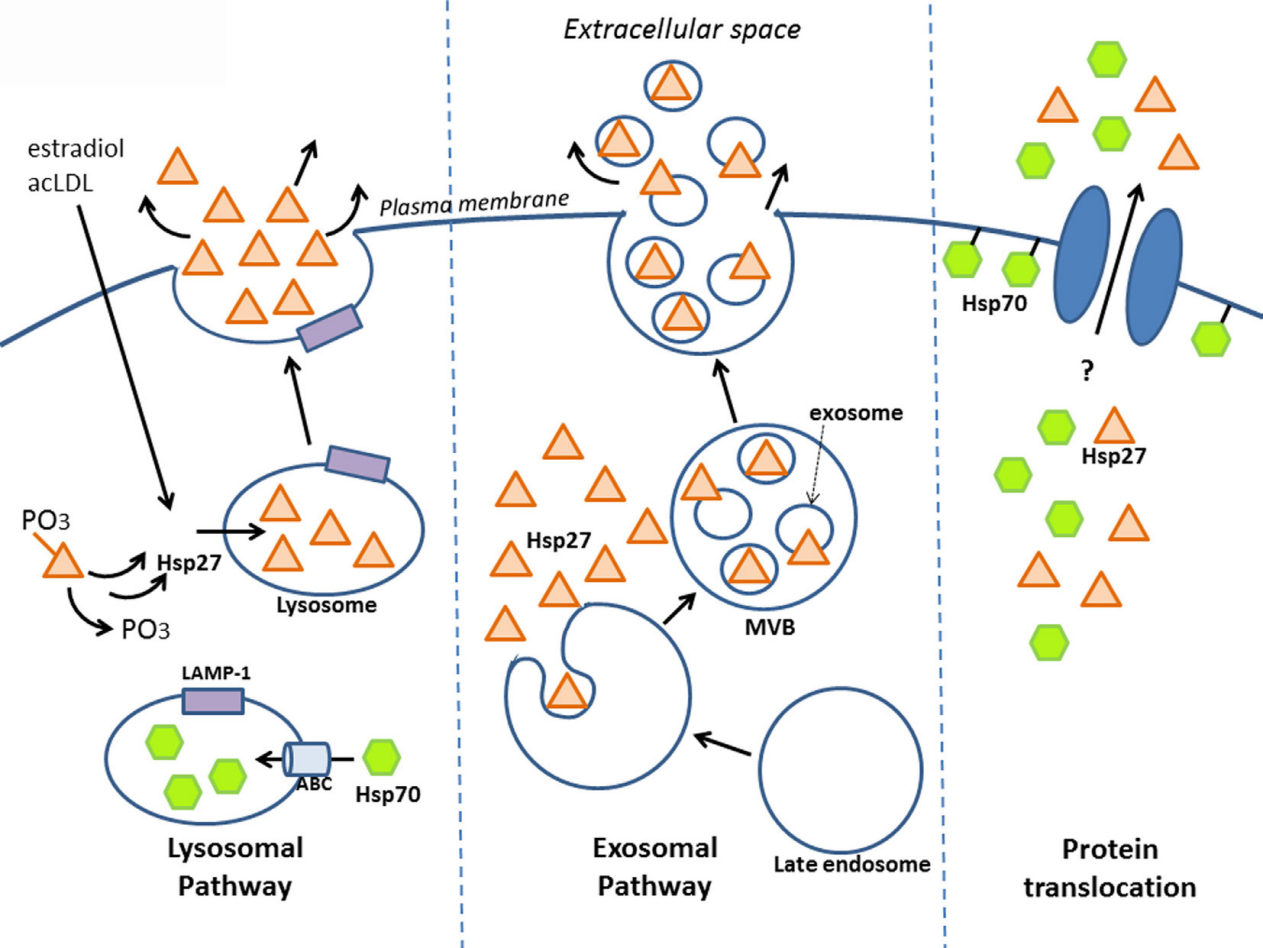
**Release of HSP27 involves non-classical secretory mechanisms**. Experimental evidence suggests that HSP27 exits cells *via* the *endolysosomal pathway* and through *exosomes*. Localization of HSP27 to lysosomes (65) may involve the dephosphorylation of two serine residues at amino acids 15 and 82 (74); meanwhile, HSP70 appears to enter lysosomes with the aid of ABC transporters spanning the lysosomal membrane (75). Although direct protein translocation accounts for the unconventional secretion of FGF-2 (76, 77), it remains to be determined whether this mechanism plays a part in HSP27 extracellular release. Like FGF-2, HSP70 can interact with lipids as well as integrate into lipid membranes (7, 46, 78–81), and it also appears that HSP70 can integrate into artificial lipid bilayers forming channels.

Secreted proteins typically exit eukaryotic cells *via* the endoplasmic reticulum–Golgi network (known as the “classical secretory pathway”). Such proteins contain an N-terminal signal peptide that marks them for secretion – which interestingly is absent in other types of secreted proteins, including HSPs. Several groups have shown that despite this, HSPs can still be released – for example, early experiments that pharmacologically blocked the classical pathway still resulted in HSP secretion (in this case, HSP70) (46). Although HSP release was proposed to be a consequence of passive transport, i.e., necrosis (68), secretion can also occur independent of cell death, implying an active transport process that involves alternative, “non-classical” secretory mechanisms (46, 69–71). Indeed, some secretory proteins lacking the signal peptide for the classical pathway have since been identified, including interleukin (IL)-1a, IL-1b, and fibroblast growth factor (FGF)-2, and increasing evidence indicates that these proteins along with some HSPs are released by non-classical secretory pathways (7, 72, 73). Although there is still much mechanistic information to uncover regarding how HSP27 is secreted, this review will discuss a few key findings indicating that it may leave the cell *via* lysosomes and/or exosomes and bring up the possibility of direct protein translocation (Figure 1).

### Endolysosomal Pathway

Known as the recycling units of the cell, lysosomes are vesicular organelles containing enzymes that break down biomolecules (proteins, lipids, nucleic acids, carbohydrates) into their basic components. In addition to their role in the degradation of cellular waste, lysosomes can also serve as storage compartments for proteins targeted for secretion. These “secretory lysosomes” have been found in blood cells, among other cell types, and share similarities with conventional lysosomes, including an acidic pH interior (82). Utilization of this alternative secretion pathway has been observed in the case of HSP70. In one study using prostate carcinoma cell lines, lysosomal fractions contained more HSP70 following heat shock, and conversely, treatment with the lysosomal inhibitors, methylamine, and ammonium chloride prevented heat shock-mediated release of HSP70 into the culture media – which together suggest that the lysosome is involved in secretion (75). It is likely that HSP70 enters the lysosomal interior *via* ATP-binding cassette (ABC) transporters, since inhibition with glibenclamide, a general ABC transporter inhibitor, and DIDS, a specific ABCA1 inhibitor, reduced HSP70 release (75). As well, its release was associated with the relocalization of lysosomal markers, typically found in the lysosomal interior to the exterior cell membrane leaflet (75, 83). Taken together, these findings indicate that HSP70 may exit cells *via* secretory lysosomes, resulting in their fusion to the cell membrane. Similar findings were seen in cultured primary human peripheral blood mononuclear cells (PBMCs), whereby HSP70 secretion was significantly inhibited by the lysosomal inhibitor, methylamine, but not by brefeldin A (a blocker of transport from the ER to Golgi) (70). Furthermore, the presence of HSP70 in the lumen of lysosomes has been reported by another group (84).

What is the evidence that HSP27 uses the endolysosomal pathway? Findings from our laboratory have shown that upon treatment of macrophages with estradiol or acetylated low-density lipoprotein (ac-LDL) – conditions that stimulate HSP27 secretion – there was increased co-localization of HSP27 with the lysosomal markers, LAMP-1, and lysotracker (65). A later study yielded new insight into how HSP27 could be released through lysosomes (74). Their experiments utilized two types of HSP27 mutations, a constitutively phosphorylated mimic (S15D/S82D) and a non-phosphorylatable form (S15A/S82A), which were linked to GFP in order to visualize cellular localization in endothelial (HUVEC) cells. Interestingly, it was observed that the S15A/S82A mutation co-localized more frequently with the lysosomal marker, LAMP-1, using fluorescence microscopy. Furthermore, using His-tagged HSP27 mutants expressed in HUVEC cells, the non-phosphorylatable HSP27 was once more found at higher levels in cell culture media compared to S15D/S82D, as assessed by Western blotting for the histidine tag. These findings suggest that in order for HSP27 to localize to the lysosome (for subsequent secretion), these two serine residues must first be dephosphorylated (Figure 1).

### Exosomal Pathway

Exosomes are the most commonly accepted vehicle for HSP release (69). This type of secretory vesicle is derived from a multistep process that first involves the internalization of the plasma membrane to form endosomes, which then develop into late endosomes. Late endosomal membranes are invaginated within, forming smaller vesicles (exosomes) that measure 40–100 nm in diameter and contain cytoplasmic components. These complex structures, referred to as “multivesicular bodies” (MVBs), fuse to the cell membrane, releasing exosomes into the extracellular space (Figure 1) (69, 85). Numerous findings have indicated the presence of several HSPs (HSP70, HSP60, HSP90) in exosomes, with localization observed at both the exosomal membrane (conceivably, facilitating interactions with cell surface receptors on target cells) and lumen (implying that for HSPs to have an effect on target cells, exosomes will have to burst to release their contents) (7, 86–90).

There is mounting evidence indicating that HSP27 is found in exosomes (Figure 1). HSP27 has been detected in exosomes derived from a variety of cell and tissue types, including keratinocytes, platelets, cancer cells, saliva, thymus, and urine, making the exosomal pathway a compelling system for HSP27 export (http://exocarta.org/gene_summary?gene_id=3315). Experiments in B-lymphoblastoid cells also demonstrate the presence of HSP27 in exosomes (90). Here, basal levels of HSP27, HSP70, and HSP90 localized to exosome fractions as assessed by Western blotting – an effect that increased with duration of heat shock treatment. When isolated exosomes were coupled to beads for flow cytometry analysis, positive staining for exosomal surface markers (CD81 and MHC class I), but not HSPs, were noted, implying that these HSPs were not expressed on the exosomal surface. However, when the exosome–bead complexes were solubilized by boiling in SDS (disrupting the exosomes) and total protein analyzed by Western blotting, elevated levels of HSP70 were detected in exosomes from heat-shocked cells compared to controls. These findings indicate that disruption of the exosomal membrane was required in order to detect HSP70, implying its localization within the exosome. Unfortunately, experiments detecting HSP27 after exosomal disruption were not presented in this study, thus it could not be confirmed whether the absence of HSP27 in the exosomal membrane points to a luminal presence. Results from our laboratory also support that exosomes may be one system involved in HSP27 release, since the treatment of human macrophages with an exosomal inhibitor, dimethyl amiloride (DMA), substantially decreased extracellular HSP27 levels (66). More recently, HSP27 was detected in exosomal fractions originating from primary cultures of rat astrocytes (91). Consensus has yet to be achieved with regards to the precise location of HSPs in exosomes. The suggestion of a luminal localization for HSP70 (90) is in contrast to results in macrophages infected with two different viruses (88, 92) and in blood cells after ischemic preconditioning (87), which indicate the presence of HSP70 in the exosomal membrane. Differences in exosomal localization is likely determined by the type of HSP, the cell from which exosomes originate, and the kind of stimuli triggering exosomal release (e.g., heat shock vs. viral infection). Another aspect of the exosomal pathway that is worth further exploration is determining the sorting signal that directs HSPs to populate exosomes. In the case of HSP60, for example, it appears to be ubiquitinated in exosomal fractions (86), suggesting that ubiquitinylation may signal sorting to exosomes. Whether this applies to HSP27 is currently unknown.

### Direct Protein Translocation

A final, intriguing possibility for HSP27 export may involve direct protein translocation across the plasma membrane, as has been proposed for FGF-2 (73, 93). Exit of HSPs from cells could be mediated by transmembrane proteins, like ABC transporters (also located on cell membranes), which, as already mentioned, are instrumental in transporting HSP70 in the lysosomes for secretion (75, 83). Another hypothetical way for HSPs to leave the cell is by directly associating with the cell membrane, which could then somehow facilitate their extracellular release. In fact, such lipid interactions have been observed with HSP70 (Figure 1) (46, 78–80), and it also appears that HSP70 can integrate into artificial lipid bilayers forming channels (7, 81). Despite the lack of evidence supporting the direct release of HSP70 (and other HSPs) after insertion or association into lipid membranes, it is still possible to consider direct membrane translocation of HSPs as a mechanism for exit from cells by looking to the cellular export paradigm utilized by FGF-2.

Over the years, the Nickel research group has systematically delineated the various steps involved in the export mechanism for FGF-2 (76, 77). According to their findings, FGF-2 localizes to the inner leaflet of the cell membrane *via* interactions with lipids, and after phosphorylation by Tec kinase, is then primed to oligomerize and integrate into the membrane, forming a transient pore structure, which itself is speculated to disassemble into FGF-2 monomers during extracellular release (76). Subsequent results highlight the importance of two surface cysteines unique to FGF-2 (notably absent in other FGF family members) that mediate disulfide bond formation with another FGF-2 monomer, forming dimers that then assemble into hexamers – the final FGF-2 structural complex that is inserted into the membrane (93). Since mutations of these two cysteines abrogate FGF-2 secretion, it appears that disulfide formation between cysteines facilitates the insertion of FGF-2 oligomers into the cell membrane and its subsequent release (93). Perhaps, similar mechanisms are involved with HSP export. FGF-2 and HSP70 have both been observed to interact with lipids and integrate into membranes. In addition, HSP27 also shares a key feature with FGF-2 – both contain unique cysteine (C) residues that happen to be missing in members of their respective protein families. In HSP27, this unique cysteine residue, C137, located in the α-crystallin domain, also mediates dimerization with other HSP27 monomers, however the consequence of dimerization differs in that disulfide formation between opposing cysteine residues modulates HSP27 function during oxidative stress (94). In reducing conditions, cysteines are directly opposite each other at the dimer interface, while under oxidative conditions, a disulfide bond forms, altering the structure of HSP27’s α-crystallin domain, leading to increased exposure of hydrophobic residues and promoting chaperoning activity (95–97). Although C137 is thought to be critical in mediating HSP27’s chaperoning activity during oxidative stress (i.e., disulfide bond can more easily bind to misfolded proteins that arise from oxidative stress), it will be interesting to see if mutation of this cysteine can alter secretion of HSP27, as has been observed with FGF-2. Another issue to address is whether HSP27 can interact with membrane lipids, like HSP70 (98–100). There is much to be discovered before ruling out that HSP27 could, in part, be exported from cells *via* direct protein transport (Figure 1).

## Signaling of Extracellular HSP27

Once outside the cell, what does extracellular HSP27 do? Most evidence suggests that, like other HSPs, HSP27 serves as a signaling molecule released by cells, which bind to surface receptors on distant cellular targets, such as endothelial and immune cells (7, 101, 102). Thus far, it appears that HSP27 can function outside the cell as (i) an uncomplexed, extracellular protein, (ii) part of a larger protein complex, for example, *via* interactions with AAB in the serum, and (iii) molecular cargoes located on the surface or inside exosomes. This review will focus on how recombinant extracellular HSP27 modifies receptor-mediated signaling in target cells and will only briefly comment on its effects as part of antibody complexes and exosomes.

**Figure 2:**
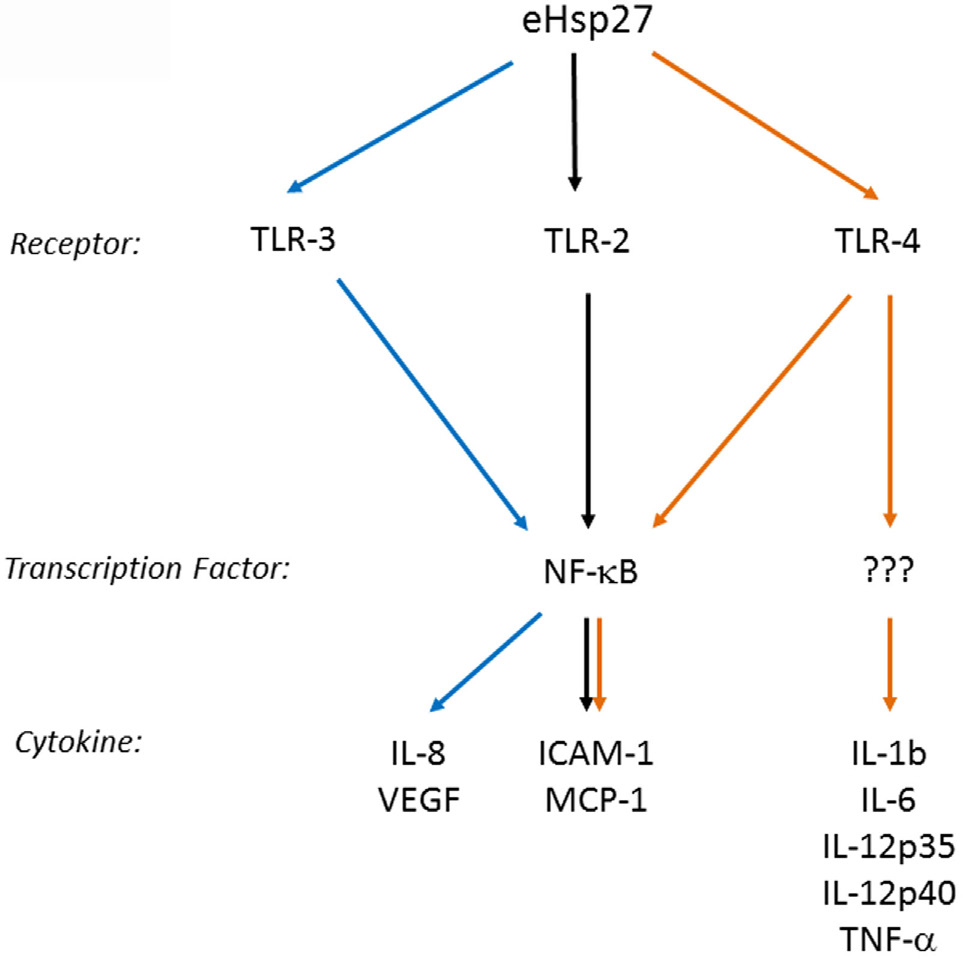
**Extracellular HSP27 (eHSP27) can trigger TLR signaling, leading to NF-κB activation and cytokine upregulation (64, 103–106)**.

Several cell membrane receptors have been identified for eHSPs, including CD91, CD40, CD36, CD14, toll-like receptors (TLRs), and scavenger receptor-A (SR-A) (8, 102). HSP27 has also been shown to interact with TLRs, as well as other types of receptors, altering signaling mechanisms in a variety of cell types and conditions (Figure 2). For example, HSP27 treatment of *human macrophages* promoted NF-κB transcriptional activation as well as protein expression and secretion of the NF-κB-dependent cytokines, GM-CSF and IL-10 – the latter important in anti-inflammatory responses (103). NF-κB activation by HSP27 was also observed in *mouse coronary endothelial cells*, occurring *via* interactions with TLR-2 and TLR-4 and resulting in the upregulation of intercellular adhesion molecule-1 (ICAM-1) and monocyte chemoattractant protein-1 (MCP-1) (64). In *human coronary endothelial cells*, HSP27 also upregulated ICAM-1, MCP-1, as well as IL-6 and IL-8, although the receptors mediating the response in human cells were not identified (64). In contrast, TLR-3 was implicated as the receptor utilized by HSP27 in *human microvascular endothelial cells*, leading to NF-κB activation and secretion of IL-8 and vascular endothelial growth factor (VEGF) (104). The effect of HSP27 on VEGF was corroborated in *endothelial progenitor cells (EPCs)*, although this study did not address which receptors play a part in VEGF secretion (105). HSP27 increased secretion of IL-6 in *bone marrow-derived dendritic cells*, as well as TNF-α, IL-1β, IL-12p35, and IL-12p40, which partly occurred through TLR-4 signaling (106). In *primary human monocytes*, release of IL-10 by HSP27 involved phosphorylation of p38 and MAPKAPK-2, while increased TNF-α levels were attributed to the activation of both p38 and ERK1/2 signaling pathways (107). HSP27 also interacts with estrogen receptor-β (ER-β) (108, 109). Altogether, these findings implicate extracellular HSP27 in (i) immune signaling (*via* activation of TLR signaling pathways), leading to cytokine production and modulation of the immune response, (ii) cellular migration (due to the upregulation of ICAM-1 and VEGF), and (iii) cellular proliferation (through its interactions with ER-β).

Extracellular HSP27 has also been found as part of larger macromolecular complexes. For example, our laboratory’s attempts to develop a more sensitive method for measuring HSP27 in serum samples led to the serendipitous discovery that this protein is complexed to AAB, which effectively shields the detection of serum HSP27. *In vitro* experiments later showed that AAB could potentiate the signaling effects of HSP27 (67). Another example of HSP27 being part of a larger complex is its presence in exosomes. Exosomes carrying HSP27 (either on the surface or in its interior) could then interact with target cells through receptor interactions leading to endocytosis, or by simple membrane fusion that is independent of receptors (69). Precisely how exosomes cargoing HSP27 affect target cells is unknown; however, it may instigate a variety of effects depending on the exosomal source and downstream cellular target. This has been observed with HSP70, whose expression on exosomal surfaces has been found to (i) trigger TLR-4 signaling leading to myocardial protection (87) and (ii) stimulate natural killer (NK) cells, increasing its cytolytic activity against HSP70-membrane positive tumors (110). Thus, by interacting with receptors on both immune and endothelial cells, leading to the differential release of cytokines and growth factors, extracellular HSP27 is a potentially important modulator of the immune response – an important process that contributes to atherosclerosis.

## HSP27 in Atherosclerosis

Atherosclerosis is a chronic cardiovascular condition that is marked by the progressive accumulation of inflammatory scar tissue containing lipid deposits, calcification, and necrotic debris within the walls of arteries that can lead to the formation of unstable plaques at risk of rupture. Plaque rupture is unpredictable and is the final phase that leads to a reduction in blood flow, ischemia in the distal vascular bed, and clinical symptoms or events (e.g., heart attack and/or death). Although the etiology of atherosclerosis was primarily thought to originate from a lipid/cholesterol imbalance (be it genetic or environmental), it appears that atheroprogression is a complex, multifactorial process that is, in part, due to coordinated immune responses. Recently, several lines of evidence support the emerging role of HSP27 in modulating atheroprogression, by altering the inflammatory components of atherosclerotic plaques.

Although evidence of cardiovascular disease has been traced back to thousands of years, with various prehistoric populations displaying signs of arterial plaque (111), it is only within the past 100 years that this condition has emerged as the leading cause of mortality in the world. Epidemiological studies from the twentieth century have indicated that women are at lower risk for developing cardiovascular diseases compared to men; however, this bias toward the female sex disappears after menopause, implicating female hormones as protective factors against CVD (112). Consequently, various clinical trials conducted in the 1970s–2000s sought to test whether the replacement of ovarian hormones could protect menopausal women from CVD. Unfortunately, the conclusions of these trials indicate that hormone replacement therapy not only failed to protect women from CVD but also increased side effects as well as CVD risk (113, 114). Although hormone replacement therapies were ineffective against CVD, the question of how the presence of female hormones protect against CVD in premenopausal women remains. Given that HSP27 was initially identified as an estrogen-inducible gene (10, 11), it was hypothesized that HSP27 is one important downstream “foot soldier” of estrogen signaling that mediates cardiovascular protection (108).

There is a precedent in establishing a link between other HSPs and cardiovascular disease. For example, some studies correlate HSP60 with atherosclerosis severity and suggest that it could serve as a biomarker (115–117), while others associate HSP70 serum levels with low CVD risk (118, 119). HSP27 was first implicated in atherosclerosis more than 10 years ago, when a proteomic screen comparing levels of released proteins noted lower levels of HSP27 secretion from human atherosclerotic arteries compared to normal arteries (60). Indeed, HSP27 secretion and expression in atherosclerotic human coronary arteries (108, 120) decreases with increasing plaque progression, complexity, and instability. Conversely, areas of normal-appearing atherosclerotic arteries display increased HSP27 expression compared to plaque-filled areas from the same vessel (61). Three independent studies confirm the reduction in secreted HSP27 levels in atherosclerosis patients. The first study involving small patient cohorts (28 patients vs. 12 controls) noted significantly lower HSP27 serum levels in CVD patients compared to healthy controls (60). Two later studies in larger patient cohorts (76 patients vs. 53 controls; 80 patients vs. 80 controls) validated this finding, with CVD patients again displaying lower levels of serum HSP27 (62, 63). Moreover, our laboratory also demonstrated that in patients diagnosed with coronary atherosclerosis (>50% stenosis by angiography), lower levels of HSP27 was predictive of subsequent major clinical events (i.e., heart attack, stroke, cardiovascular death) over a 5-year period (63). However, in a prospective, nested, case–controlled study involving initially healthy female participants who later developed cardiovascular disease, baseline HSP27 plasma levels are inversely associated with age but neither significantly associated with other established cardiovascular risk factors nor with future cardiovascular events (121). These seemingly disparate findings regarding HSP27’s potential as a marker in predicting cardiovascular events can be reconciled if one considers that all of the female subjects studied in the nested case–control series were, on average, 61 years of age at baseline and beyond menopause. Therefore, one would expect relatively low serum levels of HSP27 in both the controls and later CVD event patients at baseline. Moreover, at this age, coronary atherogenesis may have already reached a moderately advanced stage.

Hence, the results to date reveal that plasma HSP27 levels may indeed be a potential marker for atherosclerosis and predictive of adverse cardiovascular events. With further refinements in the HSP27 serum assay that will remove the confounding presence of HSP27 AAB, we are confident that this conclusion will be further validated. Additionally, diminished expression of HSP27 in atherosclerotic plaques compared to vessels free of disease further highlights the association between augmented HSP27 levels and protection against atherosclerosis.

Some investigators suggest that extracellular HSP27 may be subject to protease degradation that is more abundant in atherosclerotic compared to normal arteries. Indeed, it has been shown that plasmin is upregulated in atherosclerotic plaques and can cause the degradation and aggregation of recombinant HSP27 (rHSP27) as well as HSP27 endogenously secreted from arterial explants – findings reversed by aprotinin, a specific plasmin inhibitor (122). Other enzymes that are more abundant in vulnerable atherosclerotic plaques due to a proteolytic imbalance, like matrix metalloproteases (MMPs) (123), can also interact with (124, 125) and possibly degrade HSPs (126). Another idea to consider is that HSP27 (as well as other HSPs) could function as “danger signals” – highly abundant self-proteins normally found intracellularly in healthy tissues, which are released into the extracellular space when tissues are damaged, infected, or stressed. Such danger signals are capable of activating antigen-presenting cells (APCs) to initiate the adaptive immune response (127). Stress and damage associated with atheroprogression could thus release more HSP27 from cells into the circulation, resulting in the following possibilities: (i) less HSP27 expression in cells as plaque accumulates, (ii) proteolytic cleavage and degradation of extracellular HSP27, (iii) increased susceptibility to apoptosis of cells that have secreted HSP27, and (iv) activation/modulation of the immune response. Although the link between reduced HSP27 levels in the serum and atherosclerotic vessel walls is strong, it remains unclear whether HSP27 levels are a secondary feature of atherosclerosis or the stress response during atherogenesis, or if HSP27 plays a causal role in directly modulating vessel wall homeostasis.

### Immunological Contribution in Atherosclerosis

In order to understand how HSP27 is involved in atheroprogression and whether it could protect against development of CVD, the various stages and key cellular players involved in atherosclerosis must first be highlighted. Atherosclerosis is characterized by the development in arteries of lesions or plaques containing lipids, inflammatory markers, and dead cells surrounded by a fibrotic cap structure. Some areas of the vasculature are more susceptible to plaque accumulation. For example, branch points in the circulatory tree experience altered laminar blood flow that could lead to atherosclerosis-promoting changes (128), including endothelial dysfunction, accumulation of oxidized low-density lipoproteins (ox-LDL), alterations in matrix protein composition, and accumulation of proteoglycans, which increase ox-LDL accumulation at the cellular level (129). Plaque accumulation can impair blood flow, causing health complications, and if left alone, can escalate to the grave consequence of plaque “rupture,” exposing pro-thrombotic (pro-clotting) material from the plaque to the blood and causing occlusion of the artery. These local arterial blood clots deprive nearby cells of oxygen, leading to cell death and various adverse outcomes (depending on the rupture site), including heart failure, stroke, renal impairment, and/or hypertension (129).

**Figure 3:**
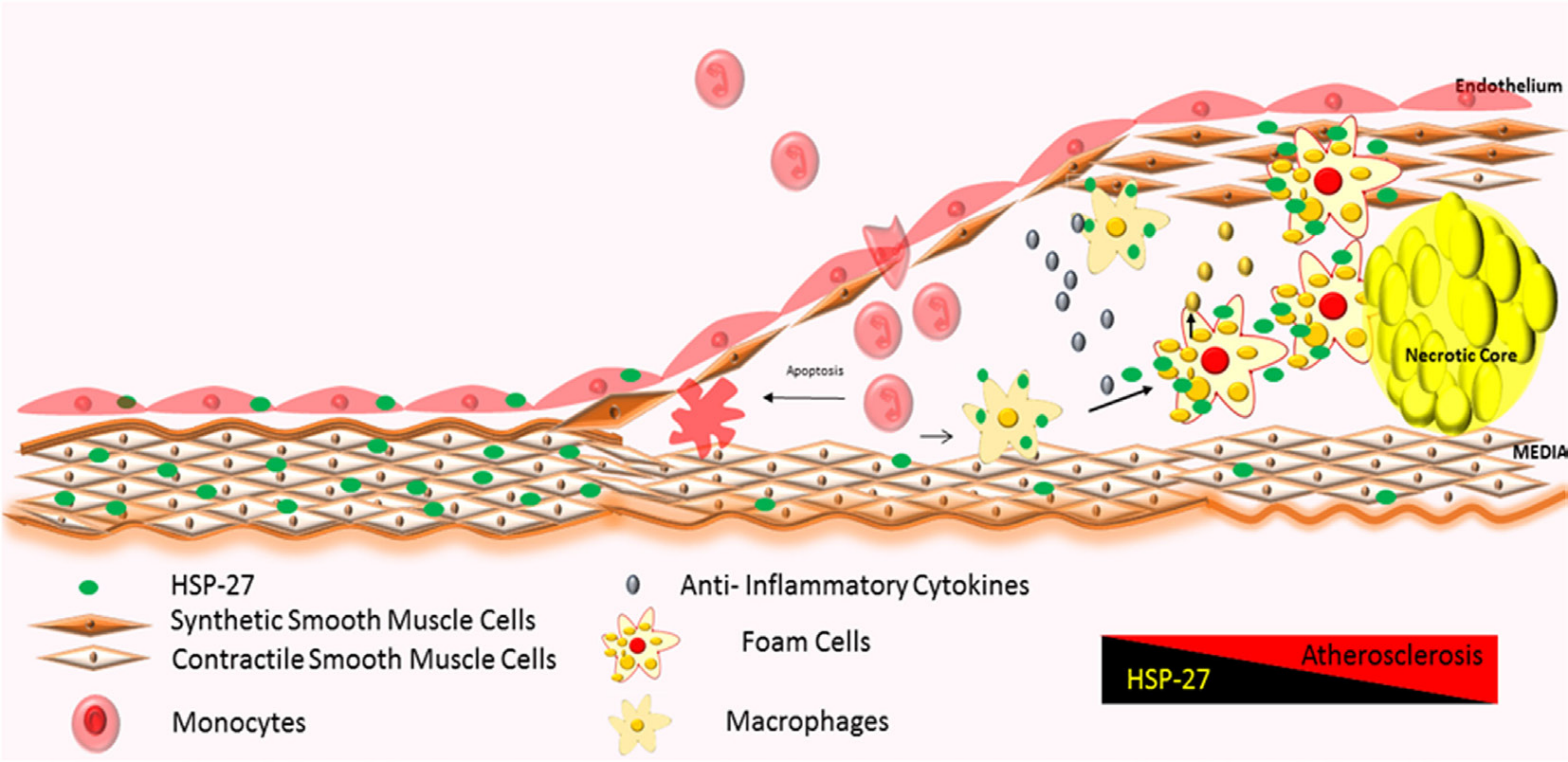
**Atherosclerotic plaque progression: severity of atherosclerosis correlates with increased infiltration of monocytes and their local activation into macrophages and foam cells, further promoting the inflammatory process (128, 129)**. HSP27 is expressed in normal areas of arteries, with reduction in expression in areas of advanced plaque (60, 61, 108, 120). Increases in HSP27 expression have also been noted in the fibrous cap and media (122).

The first stage of plaque formation involves the development of “fatty streaks.” This gradual process originates in endothelial cells lining the inner arterial wall (“intima”) and involves (1) the uptake and accumulation of circulating oxidized lipids and low-density lipoprotein (LDL), (2) expression of adhesion molecules, and (3) chemokine release, resulting in the recruitment and deposition of immune cells (mainly lipid-containing macrophages also known as foam cells and T-cells) along the intima (130). Fatty streaks do not give rise to overt symptoms and are reversible (129). It is interesting to note that the partial attenuation of HSP27 expression starts in this stage of atherogenesis (Figure 3) (108, 131).

Over time, fatty streaks become mature atherosclerotic plaques (“atheromas”) distinguished by the following regions: (1) a core region, consisting of foam cells, lipids, immune cells, such as dendritic cells, mast cells, and B-cells (129), apoptotic cells, debris, cholesterol crystals (132–134), and proteases like plasmin (122); (2) a fibrous cap region surrounding the core, made up of smooth muscle cells (122) and a collagen-rich matrix; and (3) an interface between core and fibrous cap regions (“shoulder” region), housing mainly T-cells and macrophages (129) (Figure 3). Immunohistochemical analysis of atherosclerotic arteries obtained from patients undergoing cardiac surgery show HSP27 localization mainly in the fibrous cap and media (122); however, its abundance diminishes as the complexity and volume of the plaque increases (108).

The presence of immune cells early in plaque formation indicates the involvement of the immune response; however, it is still unclear whether this promotes or attenuates atherosclerosis. The canonical idea that atherosclerosis is primarily caused by dietary and lifestyle factors still carries weight in that the rise of cardiovascular diseases came about within the last century, coinciding with increasingly sedentary lifestyles and increased caloric and fat intake. Given that fatty streaks are found in young people, it appears to be a normal adaptive process to have an immune response generated against lipids in the arteries. However, for this response to promote atheroprogression likely involves an interplay between the presence of cholesterol (in plaques) derived from the diet and circulating immune cells that become resident in the arterial wall. Hence, the human body can be tipped into developing atherosclerosis if the source of cholesterol becomes dysregulated (dietary or genetic predisposition to the development of hypercholesterolemia) or if the immune system becomes abnormally sensitized to normal levels of lipid in the blood vessels, or both.

The immune cells primarily responsible for propagating the inflammatory response against lipids in atherosclerotic plaque are macrophages. Upon encountering lipids, particularly ox-LDL, macrophages engulf ox-LDL by binding to LDL membrane receptors. Internalization of ox-LDL then triggers a cascade of events that transform macrophages into foam cells. Foam cells also secrete pro-inflammatory cytokines that result in the recruitment and activation of additional immune cells to the lesion site – further amplifying the immune response and promoting the development of complex, advanced plaques. Our previous results have shown that HSP27 competes with LDL binding to SR-A, known to take up atherogenic lipids, as well as downregulating SR-A expression (65, 135), thus attenuating foam cell formation. Furthermore, administration of recombinant HSP27 has been shown to block the differentiation of monocytes to dendritic cells, a cell type that can also promote the pro-inflammatory process (136). Another way that HSP27 can regulate the inflammatory response involved in atherosclerotic plaque progression is through the release of GM-CSF, which we have seen in cultured human macrophages, following rHSP27 treatment (103). Depending on the existing cytokine milieu, GM-CSF appears to promote the development of the M1 macrophage lineage (137), considered to play a role in the pro-inflammatory immune response (138). HSP27 may thus favor the preponderance of one macrophage phenotype over another, thereby affecting plaque progression.

Finally, another immune cell type involved in atheroprogression (albeit to a lesser degree) are T-regulatory cells, which mediate atheroprotection by amplifying the release of IL-10, thereby resulting in immune suppression (139–142). However, the role of HSP27 in T-regulatory function during atheroprogression is uncertain (143). Mast cells, a key cell type that populates atherosclerotic plaque, are classically known to react immediately to allergens *via* cross-linking of cell surface IgE resulting in degranulation, histamine release, and synthesis of arachidonic acid metabolites, which can activate stress-signaling and pro-apoptotic pathways (144). Mast cells have been shown to release proteases that can degrade HSP27 (145, 146).

### Potential Mechanisms Involved in HSP27-Mediated Atheroprotection

**Figure 4:**
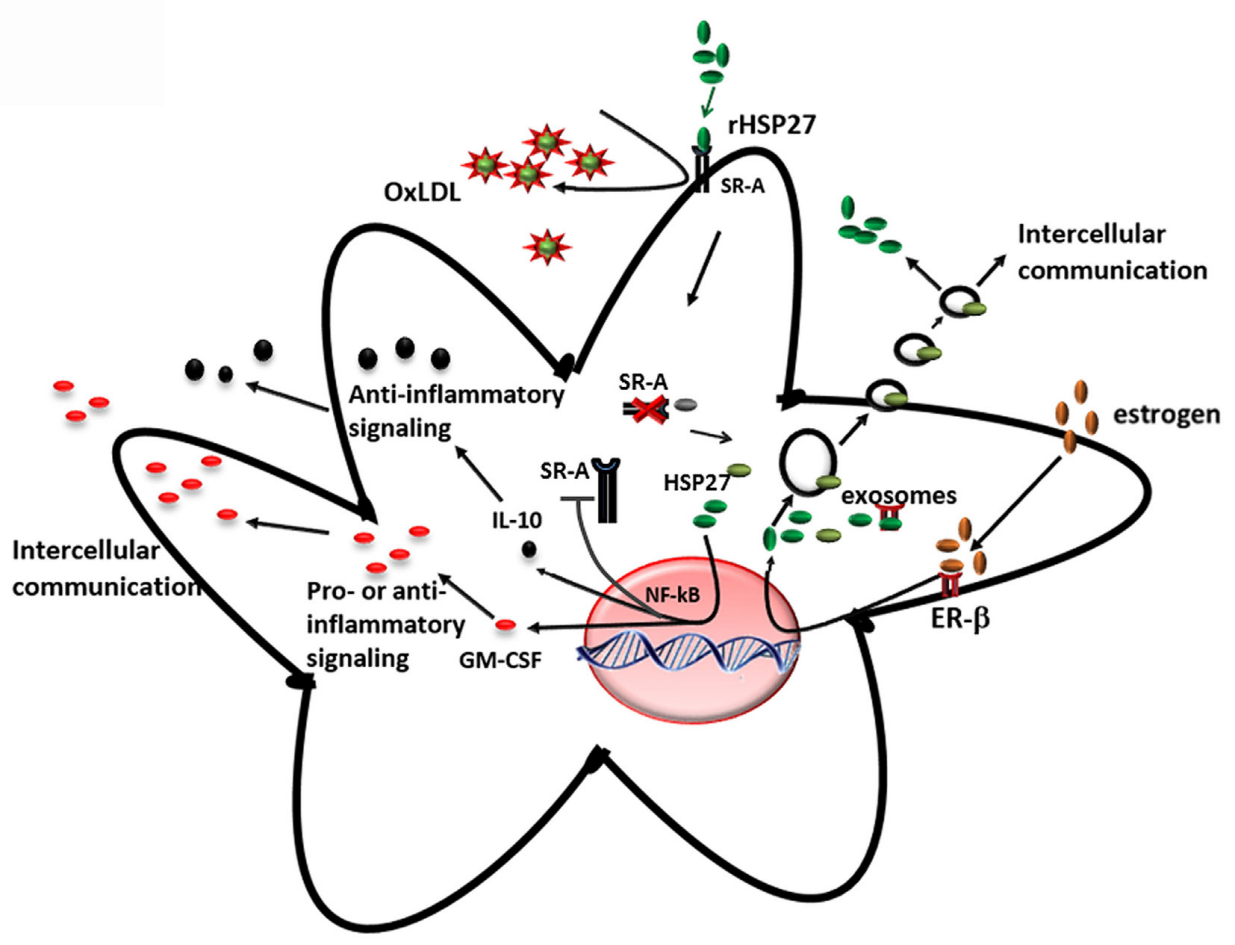
**Extracellular HSP27, whose release is stimulated by estrogen, affects macrophages by (i) reducing ox-LDL uptake and (ii) activating NF-κB, which results in increased secretion of IL-10 and GM-CSF (65, 103, 135)**.

The key findings from our research group and others showing that (i) the loss of HSP27 serum levels correlates with plaque and disease progression and (ii) HSP27 is absent at sites of mature, complex plaque (while present in normal intima from the same patient with atherosclerosis), suggest that HSP27 could be atheroprotective. To address this possibility, mice prone to atherosclerosis (ApoE^−/−^) (147) were crossed to transgenic mice overexpressing (human) HSP27 (HSP27^o/e^) (65). Circulating HSP27 serum levels were higher in HSP27^o/e^ ApoE^−/−^ mice compared to ApoE^−/−^ control mice and were inversely correlated with lesion area in both male and female mice (65, 148). More importantly, HSP27 overexpression protected mice from atherosclerosis, particularly in female mice (65) – a process that was abrogated by ovariectomy but rescued by administration of exogenous estrogens (66). Taken together, these data indicate that secretion of HSP27 into the circulation requires estrogens (66). Compared to ApoE^−/−^ mice, HSP27^o/e^ ApoE^−/−^ mice exhibited reductions in cholesterol cleft areas, macrophage infiltration, apoptotic cell number in the plaques, and free serum cholesterol. Features indicative of favorable plaque remodeling (e.g., increased smooth muscle cell and collagen content and greater vessel stiffness) were also evident (148). Administering HSP27 to atheroprone mice *via* bone marrow transplant from HSP27^o/e^ mice or recombinant HSP27 injections also reduced atherosclerotic lesions and increased plaque stability (63). These *in vivo* findings yielded several mechanistic possibilities underlying HSP27-mediated atheroprotection. First, the presence of estrogen appears to be important for HSP27’s extracellular release. Second, HSP27 could modify various processes involved in atherosclerosis, including cholesterol homeostasis/trafficking, inflammation, immune cell infiltration into and migration out of plaques, activation of macrophages to foam cells, and plaque remodeling (Figure 4).

#### Regulation of Cholesterol in Macrophages by HSP27

An interesting finding from the HSP27 overexpressing atherogenic mouse model is the reduction in cholesterol, in both plaque and serum (148), suggesting that HSP27 is involved in lipid homeostasis. One class of receptors known to bind to modified lipoproteins and facilitate their internalization in macrophages is SR-A (149, 150). It has been suggested that unregulated uptake of modified LDL in macrophages promotes the differentiation of monocyte-derived macrophages into pro-inflammatory foam cells, resulting in their accumulation in the lumen and, consequently, the narrowing and hardening of blood vessels (151, 152). *In vitro* experiments from our laboratory have shown that extracellular HSP27 affects SR-A activity *via* protein interactions on the surface of macrophages (65). This interaction reduces the binding of LDL to SR-A, and consequently, the formation of cholesterol-laden macrophages (65, 135). However, recent experimental data also support the role of HSP27 as a downregulator of SR-A protein expression (135). It has been shown that treatment of macrophages with extracellular recombinant HSP27 (rHSP27) is associated with reductions in both SR-A mRNA levels (−39%) and cell surface expression (−58%) as well as less foam cell formation – effects that occur *via* NF-κB signaling (135). Knockout of SR-A in ApoE^−/−^ atheroprone mice abrogated the protective effects (reductions in atherosclerotic lesions) of HSP27, indicating HSP27 atheroprotection appears to depend, in part, on the presence of SR-A (135). Recent experiments in our laboratory indicate that HSP27 modulates cholesterol efflux in macrophages *via* ABCA1 and ABCG1 transporters (153). Thus, HSP27 has numerous, pleiotropic effects on cholesterol trafficking, modulating both import and export of cholesterol in macrophages (Figure 4).

#### Modulation of the Inflammatory Response

Less atherosclerotic lesions in HSP27^o/e^ ApoE^−/−^ suggested that HSP27 can modulate the immune processes involved in atherosclerosis. One possibility lies in HSP27-mediated regulation of NF-κB activity in macrophages. NF-κB transcription factors regulate a vast number and diversity of gene targets, including those involved in cell proliferation, apoptosis, the cell stress response, inflammation, and both innate and adaptive immune responses (154, 155). Normally found in the cytoplasm as inactive dimers associated with IκB, NF-κB translocates to the nucleus once IκB is phosphorylated by the upstream IκB kinase complex (IKK), leading to the transactivation of numerous gene targets (154, 156). NF-κB-dependent pathways may influence the inflammatory process in atherosclerosis. Various experimental models indicate that *intracellular* HSP27 attenuates NF-κB activation and signaling. In skeletal muscle disuse-mediated atrophy, the activation of NF-κB is reversed by HSP27 (157). Cardiac-specific overexpression of HSP27 was found to be protective against LPS-induced cardiac dysfunction in mice *via* inhibition of the NF-κB pathway (158). Also, heat shock treatment, which upregulated HSP27, was thought to protect against angiotensin II-induced inflammation *via* an inhibitory effect of HSP27 on the NF-κB pathway by reducing both phosphorylated and non-phosphorylated IKK-α (159, 160). However, several studies show the opposite effect of HSP27 on NF-κB. HSP27 overexpression enhances the 26S proteasome and augments the degradation of phosphorylated IκBα. As described previously, this leads to the activation and nuclear translocation of NF-κB, which the authors suggest is responsible for HSP27’s downstream anti-apoptotic properties (161). More recently, Park and colleagues found that some pro-inflammatory cytokines, such as TNF-α, can enhance the binding of HSP27 to other NF-κB protein partners, such as IκB kinases (IKKs), and this interaction is promoted by the phosphorylation of HSP27 (162). While these studies all focused on *intracellular* actions of HSP27, whether *extracellular* HSP27 has an effect on the NF-κB pathway is starting to be explored.

Within atherosclerotic plaque, NF-κB is activated mainly in endothelial cells, smooth muscle cells, and macrophages. Based on the evidence supporting the role of HSP27 in the activation of NF-κB, and also the release of HSP27 from macrophages by estrogen (66, 135), it is apparent that extracellular HSP27 may also contribute to NF-κB signaling during atherosclerosis (Figure 4). Indeed, macrophages exposed to an extracellular recombinant HSP27 (rHSP27) consistently activated NF-κB through the degradation of IκBα. This effect was followed by a change in the transcriptional profile of some target genes coding for pro- and anti-inflammatory cytokines, such as IL-6, GM-CSF, TNF, or IL-10 (103), confirming the evidence that cytokines are implicated at various stages of atherosclerosis. Moreover, it was also shown that HSP27 attenuated ac-LDL-induced release of the pro-inflammatory cytokine, IL-1β, while increasing secretion of the anti-inflammatory cytokine, IL-10, in macrophages (65). Previous findings in primary human monocytes support this anti-inflammatory role, as extracellular rHSP27 significantly induced IL-10 production and only minimally upregulated TNF-α (107).

#### Protection of the Endothelial Vasculature from Oxidative Stress and Apoptosis

Extracellular HSP27 may also protect the endothelium from various stress factors inherent in the vasculature. The vessel wall and subendothelial space are continually exposed to stress from hemodynamic strain, as well as damaging factors within the blood itself (i.e., pro-inflammatory molecules, pathogens, carcinogens, and products of oxidative stress). Reactive oxygen species (ROS) can modify LDLs in the blood, turning it into ox-LDL, which has been shown to activate NADPH oxidase and xanthine oxidase, thereby inducing oxidative stress (163). Additionally, exposure of endothelial cells to LDL and ox-LDL increases the levels of the ROS species, O^2−^, and NO (164, 165). During oxidative stress, HSP27 functions as an antioxidant in cells, lowering the levels of ROS by reducing the levels of intracellular iron and raising intracellular levels of glutathione (166). HSP27’s presence in the circulation may therefore affect ox-LDL’s contribution to atherogenesis on two levels – by competing with its uptake into macrophages (Figure 4) (65) and by lowering intracellular ROS production in endothelial cells, which would then curb the subsequent oxidative modification of LDL in the blood. An additional cardioprotective effect stems from the ability of HSP27 to protect the endothelium from ischemic insult by maintaining the integrity of cytoskeletal proteins (167–171). HSP27 can also preserve the endothelial barrier by functioning as a general anti-apoptotic protein, since downregulation of HSP27 in retinal capillary endothelial cells by both cytokines and siRNA increased apoptosis (172). Thus, HSP27-based treatment strategies – which could potentially prevent LDL oxidative modification as well as protect the endothelium/accelerate its repair – may be effective in reducing lesion formation. Although the majority of the above findings related to endothelium protection involve *intracellular* HSP27, recent results from our laboratory implicate *extracellular* HSP27 in the regeneration of the endothelial barrier (105). Using primary human EPCs treated with rHSP27 and mice overexpressing HSP27 (HSP27^o/e^ mice), it was found that after vascular injury, re-endothelialization improved in both the experimental models, and neointima formation was decreased in HSP27^o/e^ mice compared to wild type – effects partly mediated through the secretion of VEGF and its paracrine effect on EPCs (105).

## Future Directions

By mediating the immunological process of atherosclerosis (Figures 3 and 4) and attenuating disease symptoms and progression, HSP27 has considerable therapeutic potential in the treatment of cardiovascular diseases. Currently, our laboratory is considering several strategies to capitalize on the atheroprotective effect of HSP27 *via* its emerging role as an immune modulator.

The first and simplest approach is to administer HSP27 to patients. Indeed, as described above, in mice prone to developing atherosclerosis, subcutaneous injections of rHSP27 reduced lesion formation and total cholesterol levels (63). Although these preclinical findings in animal models are encouraging, several questions remain in the context of its application in the clinical setting – for example, what is the best route of administration (subcutaneous vs. intravenous injection vs. oral intake)? Also, what is the most effective formulation (the complete HSP27 protein vs. an HSP27 protein fragment)? An important caveat to keep in mind as well is that the administration of HSP27 may pose risks in the case of cancer, since *extracellular* HSP27 has been shown to promote (i) the transendothelial migration of primary tumor cells (49) and (ii) the differentiation of macrophages to phenotypes that tolerate human breast cancer cell progression (48). One must therefore carefully weigh the advantages and disadvantages concerning exogenous administration of HSP27 as a therapeutic strategy in atherosclerosis.

Another approach to consider is to harness the organism’s inherent immunity when exposed to rHSP27. The discovery in our laboratory of HSP27 being complexed with AAB in the blood (thus confounding its detection through conventional ELISA kits) led to the development of a more sensitive, mass spectrometric-based detection assay, as well as mechanistic insight into how HSP27 exists/functions outside the cell. Our preliminary studies suggest that HSP27 AAB may, in fact, have salutary effects on HSP27 signaling and atheroprotection (67). The existence of AAB against HSPs in the context of atherosclerosis is not new – for example, antibodies generated against microbial HSP60 may cross-react with endogenous HSP60 expressed in the vessel lumen (molecular mimicry), enhancing inflammation and promoting atherosclerosis (173). HSP27 AAB are present in other disease states like cancer (174) and acute heart disease (175–177), although it is difficult to gage how levels correlate with disease. Higher antibody titers are associated with improved survival of breast cancer patients, but elevations also correlate with adverse conditions, such as metabolic syndrome (177) and the early phase of acute coronary syndromes (e.g., myocardial infarction and unstable angina) – although in acute coronary syndromes, HSP27 antibody levels rapidly fall after a brief spike (175). It is still unclear how HSP27 AAB are linked to CVD – one study correlated elevations with CVD (176), while other findings indicate the opposite, that HSP27 autoantibody titers (67, 178) are higher in healthy control subjects compared to CVD patients. Indeed, if the presence of HSP27 antibodies complexed to HSP27 promotes atheroprotection, immunization strategies, either active (vis-à-vis injection with an HSP27-derived antigenic peptide with or without adjuvant) or passive (administration of the antigenic peptide combined with already generated AAB), could provide exciting, new therapeutic possibilities.

Finally, there are alternative methods for boosting HSP27 function. For example, some microRNAs (“miRs,” which are 20–22 nt-long non-coding RNA molecules that regulate gene expression by binding to nascent mRNA transcripts, targeting them for degradation), which downregulate HSP27 mRNA transcripts, can be targeted for degradation by its complimentary sequence, known as “miR inhibitor” or “anti-miR” (179, 180). Proof of this principle has been demonstrated in an insulin-resistance rat model, where decreased expression of miR-1 and miR-133 increased the level of HSP27, contributing to the loss of muscle adaptability (181). Our laboratory is currently investigating which miR species are specifically upregulated in atherosclerosis and whether these alter HSP27 levels; if so, there is a potential to target such miRs with anti-miR therapy in atherosclerosis patients. There are also endogenous methods of increasing levels of circulating HSPs – for example, a huge literature already exists correlating exercise with increased serum HSP levels (182). In light of a recent Finnish study linking frequency of sauna use with reduced risk of CVD (183), it appears that subjecting the body to periods of mild to moderate heat stress may be very beneficial for the health. These sentiments – extolling the virtues of heat – are best expressed by a quote attributed to the ancient sage and physician Hippocrates: “That which drugs fail to cure, the scalpel can cure. That which the scalpel fails to cure, heat can cure. If the heat cannot cure, it must be determined to be incurable” (184, 185). By modulating atheroprogression through cross talk with the immune system, strategies that increase HSP27 levels are promising therapeutic approaches to consider.

## Author Contributions

ZB – planning and organizing structure of the review; contributed to the sections (writing): introduction, background, extracellular HSP27, HSP27 in CVD, immunological contribution, mechanisms of atheroprotection, future directions; and planning and creation of figures. VV – contributed to the sections (writing): immunological contribution; planning and creation of figures. YL – planning and organizing structure of the review; contributed to the sections (writing): background, HSP27 in CVD, mechanisms of atheroprotection; planning and creation of figures. GK – planning and organizing structure of the review; contributed to the sections (writing): HSP27 in CVD, immunological contribution, mechanisms of atheroprotection; and planning and creation of figures. DA-O – contributed to the sections (writing): mechanisms of atheroprotection. CS – planning and organizing structure of the review; contributed to the sections: background, extracellular HSP27, HSP27 in CVD. EO – planning and organizing structure of the review; contributed to the sections writing/critically reviewing manuscript, planning the structure/outline; critical review of the manuscripts; and contributed to all sections (writing/editing).

## Conflict of Interest Statement

The authors declare that the research was conducted in the absence of any commercial or financial relationships that could be construed as a potential conflict of interest.
